# Multi-Tier Validation of a Single-Lead Wearable ECG Signal-Processing Pipeline: Clinical Comparison and Algorithmic Benchmarking on Public Expert-Annotated Databases

**DOI:** 10.3390/s26185905

**Published:** 2026-09-17

**Authors:** Josip Vrdoljak, Dora Aletta Racz

**Affiliations:** 1 Laboratory for Cardiometabolic Research, University of Split School of Medicine, 21000 Split, Croatia; 2 Smart Guard Technology Plc., 1138 Budapest, Hungary; dora@smartguardtechnology.com

**Keywords:** wearable ECG, single-lead electrocardiography, signal processing, PQRST delineation, QT interval, algorithm validation, Bland–Altman, LUDB, QTDB, Lead I, wrist-worn ECG

## Abstract

Wearable single-lead electrocardiogram (ECG) devices are increasingly used for out-of-clinic rhythm monitoring, but the rigour with which their signal-processing pipelines are validated varies widely across the literature, with most published reports relying on either a single clinical comparison or a single algorithmic benchmark. We propose and apply a multi-tier validation methodology—combining clinical reference comparison, expert-annotated database benchmarking, and a deliberate morphological stress-test—and demonstrate it on the SmartGuard single-lead wearable ECG device and its signal-processing pipeline. The three tiers comprise (i) a within-subject clinical comparison in 31 healthy adult volunteers against a Bionet Cardio 7 limb-lead Lead I reference with cardiologist-confirmed measurements (a two-electrode limb configuration approximating Lead I, not a full diagnostic 12-lead ECG), (ii) algorithmic benchmarking on the Lobachevsky University ECG Database (LUDB, 200 records, expert annotations), and (iii) a robustness stress-test on the QT Database (QTDB, 103 records, hybrid expert annotations). The pipeline integrates discrete wavelet transform-based PQRST delineation with a derivative-based QRS-width refinement, a tangent-method T-wave end estimator, and median-absolute-deviation robust averaging. In the clinical tier, mean within-subject biases were negligible for heart rate, QT, QTc and RR (all *p* > 0.6) and borderline for QRS duration (+7.3 ms, *p* = 0.066), with a systematic R-wave amplitude underestimation (−0.15 mV, *p* < 0.0001). On LUDB, heart-rate and RR-interval agreement were excellent (Pearson r > 0.95) and QRS duration good (r = 0.73, MAE 13.8 ms). On QTDB, performance degraded in line with the increased morphological heterogeneity of the dataset, particularly for QT-related metrics. Despite this minimal mean bias at the population level, individual-record QT and QTc errors showed high dispersion, precluding accurate clinical QT interpretation from single wearable traces in individual patients. Moreover, the resting, healthy-adult design of the clinical tier means its findings cannot be extrapolated to arrhythmic conditions or exercising states. The protocol is described in sufficient detail to serve as a template for evaluating other wearable single-lead ECG devices.

## 1. Introduction

Wearable and portable single-lead electrocardiogram (ECG) devices have moved rapidly from consumer accessories into the clinical mainstream, offering low-burden rhythm monitoring, opportunistic arrhythmia screening, and remote cardiac follow-up [1,2,3,4]. Large prospective studies such as the Apple Heart Study (*n* ≈ 419,000) [1] and the Fitbit Heart Study (*n* ≈ 455,700) [2] have established that smartwatch-based detection of atrial fibrillation (AF) is feasible at the population scale, while validation work on Apple Watch Lead I tracings [3,4] and on the AliveCor KardiaMobile family [5] has shown encouraging agreement with reference to 12-lead ECG for selected interval and amplitude metrics. Beyond these landmark single-arm studies, the randomised eBRAVE-AF trial demonstrated that smartphone-based digital screening more than doubles the detection and treatment of atrial fibrillation compared with usual care [6]. The Huawei Heart Study, furthermore, showed that wearable-based screening can be coupled to an integrated care pathway at the population scale [7]. Reflecting this rapid maturation, the 2021 ISHNE/HRS/EHRA/APHRS collaborative statement on mobile health in arrhythmia management explicitly calls for rigorous, transparent validation of consumer ECG devices before their outputs are integrated into clinical decision-making [8]. Recent systematic reviews and meta-analyses confirm both the promise and the heterogeneity of the field: Pooled smartwatch ECG algorithms reach sensitivities and specificities near 85–95% for AF detection, but performance varies substantially across devices, populations, and analysis conditions [9,10]. Despite this progress, the quantitative outputs of single-lead wearables—heart rate (HR), RR interval, QRS duration, QT and corrected QT (QTc) intervals, and amplitude measures—must still be rigorously validated against established clinical systems and expert-annotated references before they can be confidently used in diagnostic decision-making [11,12].

How such devices should be validated is itself an active methodological question. The V3 framework—verification, analytical validation, and clinical validation—proposed for biometric monitoring technologies distinguishes between establishing that an algorithm correctly computes a metric from sensor data (analytical validation) and establishing that the metric agrees with a clinical reference in the target population (clinical validation) [13], and its recent V3+ extension adds usability as a fourth pillar [14]. Regulatory performance standards for ambulatory ECG systems, such as IEC 60601-2-47 and ANSI/AAMI EC57, similarly mandate testing against annotated reference databases with standardised reporting metrics [15,16]. In practice, however, most published wearable-ECG validation studies address only a single layer of this hierarchy—either a clinical comparison or a database benchmark—and rarely both within one transparent protocol.

The signal-processing pipeline that translates a raw single-lead waveform into clinical metrics typically comprises baseline cleaning, R-peak detection, delineation of the P–Q–R–S–T fiducial points, and extraction of derived intervals and amplitudes. R-peak detection is a comparatively mature problem dominated by descendants of the Pan–Tompkins algorithm [17], whereas accurate single-lead delineation—particularly for the onset and offset of the QRS complex and the end of the T wave—remains substantially more challenging. Wavelet-based delineation methods, most notably the discrete wavelet transform (DWT) approach of Martínez et al. [18], provide robust automatic boundary detection across multiple databases, and open-source implementations such as NeuroKit2 [19] make these methods readily reproducible. However, delineation accuracy is sensitive to lead geometry, signal-to-noise ratio, baseline drift, and morphological variability—factors that are amplified when the input is a wrist-derived single-lead signal rather than a clean 12-lead recording. T-wave end determination, in particular, has long relied on the tangent method originally proposed by Lepeschkin and Surawicz [20] and remains a critical determinant of QT/QTc precision. A reliable wearable pipeline must therefore combine well-understood reference techniques with targeted refinements—for example, derivative-based QRS-width estimation and tangent-method T-wave end detection—and must demonstrate this reliability across multiple validation tiers.

In parallel with these classical techniques, deep-learning approaches to ECG delineation have advanced rapidly. Encoder–decoder segmentation networks of the U-Net family now report F1-scores exceeding 97–99% for P, QRS, and T waves on LUDB [21], with recent architectures adding noise-robust context fusion [22] and post-processing strategies for arrhythmic morphologies [21]. Open-source delineation has also progressed beyond the DWT. A peak-prominence-based delineator introduced in 2024 achieves sensitivity and positive predictivity above 98% for all fiducial points on standard databases [23]. Notably, a recent head-to-head assessment of heuristic and deep-neural delineators on single-lead wearable ambulatory data found that optimised classical methods perform comparably to deep networks while requiring a fraction of the computational budget [24]. For an embedded wearable pipeline that must run deterministically on-device, remain interpretable for regulatory review, and be reproducible from published parent methods, a classical DWT-plus-refinement architecture therefore remains a deliberate and defensible design choice—provided its accuracy is demonstrated across validation tiers, which is the purpose of this study.

A further challenge specific to wearables is signal quality. Wrist- and finger-derived single-lead recordings exhibit lower and more variable signal-to-noise ratio than clinical Lead I, with motion artefacts, baseline drift, and electrode-contact variability degrading delineation accuracy in activity-dependent ways [25,26]. Robust per-beat aggregation and explicit stress-testing on morphologically heterogeneous recordings are therefore essential components of any credible wearable validation protocol. Quantifying performance degradation under motion is therefore a high-priority requirement for wearable-ECG validation, and is addressed as a priority item in the future-work agenda of this study (Section 4.6).

Existing validation literature on wearable single-lead ECG splits broadly into two categories. The first comprises clinical comparison studies in which a wearable Lead I trace is recorded simultaneously with, or immediately adjacent to, a reference 12-lead ECG, and pairwise differences in interval and amplitude metrics are computed [3,4,5,11,12]. Saghir et al. compared Apple Watch Lead I against 12-lead ECG in healthy adults and reported moderate-to-strong agreement for QRS and QT intervals [3]. Spaccarotella et al. extended this to QT measurement specifically, demonstrating lead-dependent agreement between smartwatch and 12-lead QT [4]. The EVALECG Cardio study compared the AliveCor KardiaMobile 6L against the standard 12-lead in unselected cardiology patients and reported strong concordance for HR, PR, QRS, and QT intervals [5]. The second category comprises algorithmic benchmarking studies that compare ECG processing pipelines against expert-annotated public databases such as the Lobachevsky University ECG Database (LUDB) [27]—200 ten-second 12-lead recordings with cardiologist-verified P, QRS, and T boundaries—and the QT Database (QTDB) [28], with manually reviewed waveform boundaries on heterogeneous longer recordings. More recent clinical comparisons have broadened this evidence base. The BASEL Wearable Study evaluated five direct-to-consumer devices against physician-interpreted 12-lead ECG in a real-world cardiology cohort [29]. A 2024 systematic review of the KardiaMobile 6L for QT monitoring found mean QTc differences within ±10 ms of the 12-lead reference [30]. Validation has been extended to paediatric populations, where agreement was excellent for heart rate but only moderate for QRS and QTc [31]. While each of these designs is informative in isolation, comparatively few published studies combine both—that is, paired clinical comparison of the device against a contemporary reference standard and algorithmic benchmarking against expert-annotated databases—into a unified, multi-tier validation framework that can be transparently reported and replicated for other wearable devices.

In this study, we present such a multi-tier validation protocol applied to the SmartGuard single-lead wearable ECG device. The protocol combines (i) a within-subject clinical comparison in 31 healthy adult volunteers, with simultaneous matched-posture recordings against a Bionet Cardio 7 limb-lead Lead I reference and with cardiologist-confirmed manual measurements; (ii) algorithmic benchmarking on LUDB (200 records, 12-lead, cardiologist-annotated), used to characterise interval delineation accuracy under cleaner conditions; and (iii) a stress-test on QTDB (105 records, hybrid expert annotations), used to probe robustness on longer and morphologically heterogeneous recordings. The signal-processing pipeline integrates DWT-based delineation [18,19] with derivative-based QRS-width estimation derived from the Pan–Tompkins family [17] and a tangent-method T-wave end detector [20], together with median-absolute-deviation robust averaging to reduce the influence of outlier beats. Statistical analysis is reported using paired t-tests, Bland–Altman limits of agreement [32], Lin’s concordance correlation coefficient [33], and per-record Pearson correlation against expert annotations. In V3 terms [13], Tier 1 constitutes clinical validation against a contemporary reference device, while Tiers 2 and 3 constitute analytical validation under clean and deliberately adverse conditions, respectively.

The contribution of this paper is therefore twofold. First, we describe a multi-tier validation methodology—combining within-subject clinical comparison, expert-database benchmarking, and a deliberate morphological stress-test—and demonstrate it in sufficient detail to serve as a transparent template for evaluating other wearable single-lead ECG devices, with the goal of standardising and improving the rigour of future wearable-ECG validation studies. Second, we apply this methodology to the SmartGuard single-lead wearable ECG pipeline and report per-tier validation performance against clinical and expert references, identifying both the strengths (HR, RR, and global QT/QTc bias) and the residual limitations (R-amplitude calibration, QRS-boundary definition, and T-wave end detection on heterogeneous recordings) of the current pipeline. The remainder of this manuscript is organised as follows. Section 2 describes the SmartGuard device and the signal-processing pipeline at a stage-by-stage level, with parameter choices and citations to the parent methods. Section 3 details the validation framework, datasets, and statistical analysis plan. Section 4 reports per-tier results. Section 5 discusses agreement, residual limitations, and avenues for algorithmic improvement.

In summary, the main contributions of this work are the following:

- A three-tier validation methodology that combines within-subject clinical comparison, expert-annotated database benchmarking, and a deliberate morphological stress-test, mapped onto the V3 framework and reported in sufficient detail to be replicated for other wearable single-lead ECG devices (Table 1);
- A fully documented single-lead processing pipeline that integrates published parent methods with two custom refinements—a derivative-based QRS-width estimator and a tangent-method T-wave end detector—plus MAD-based robust averaging, suitable for deterministic on-device execution (Section 2.2);
- Quantitative per-tier evidence: near-zero group-level bias for HR, RR, QT, and QTc in the clinical tier; LUDB delineation accuracy competitive with published wavelet delineators; and an explicit characterisation of degradation modes on QTDB;
- Identification of the pipeline’s residual failure modes—R-amplitude calibration, QRS-boundary convention, and T-wave end detection on heterogeneous morphologies—together with concrete algorithmic remedies for each.

**Figure 1:**
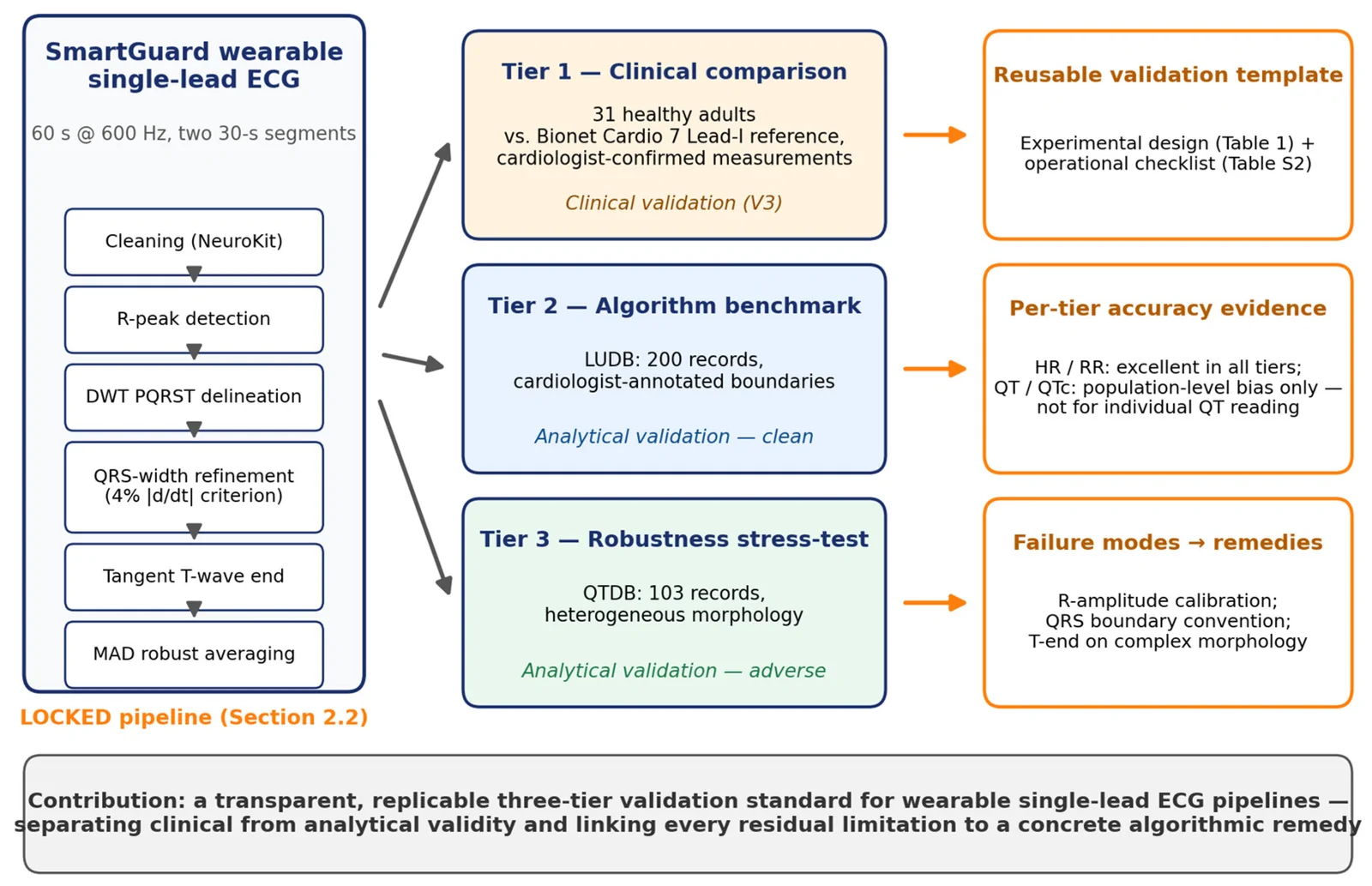
Graphical overview of the study’s design and contributions. The locked SmartGuard signal-processing pipeline (left; Section 2.2) is evaluated by three complementary validation tiers (centre; Section 2.3), each mapped to its role within the V3 framework—clinical validation (Tier 1) and analytical validation under clean (Tier 2) and deliberately adverse (Tier 3) conditions. Each tier feeds one of the study’s three outputs (right): a reusable validation template with an operational checklist, per-tier accuracy evidence, and an explicit mapping from residual failure modes to concrete algorithmic remedies. Grey arrows indicate the flow of the locked pipeline output into the three validation tiers; orange arrows link each tier to its principal output. Navy outlines denote the device and pipeline stages; the beige, blue, and green tier backgrounds distinguish the clinical, clean-benchmark, and stress-test tiers, respectively; orange outlines mark the study outputs.

Figure 1 summarises this study graphically. A locked single-lead processing pipeline is confronted with three complementary validation tiers—each mapped to its role in the V3 framework—yielding the three outputs that constitute the contributions listed above: a reusable validation template, per-tier accuracy evidence, and an explicit mapping from residual failure modes to algorithmic remedies.

## 2. Materials and Methods

This section is organised around the two elements that constitute the proposal of this paper. The first is a compact single-lead ECG signal-processing pipeline (Section 2.1 and Section 2.2) that combines well-established parent methods—NeuroKit2 cleaning, Pan–Tompkins-family R-peak detection, and DWT-based PQRST delineation—with two custom refinements designed specifically for wearable signals: a derivative-based QRS-width estimator and a tangent-method T-wave end detector, followed by median-absolute-deviation robust averaging. The second is a three-tier validation design (summarised in Table 1 and detailed in Section 2.3) in which the same locked pipeline is confronted with (i) a clinical reference device, (ii) a clean expert-annotated database (LUDB), and (iii) a deliberately heterogeneous stress-test database (QTDB). Representative waveforms from both databases are illustrated in Section 2.3. The remainder of this section details each component in turn.

### 2.1. Device Hardware and Signal Acquisition

The index device evaluated in this study is the SmartGuard portable smart ECG device (Smart Guard Technology Plc., Budapest, Hungary), deployed as a wrist-worn ECG device and configured to record a single-lead, Lead I-equivalent electrocardiogram. The acquisition circuit is closed by contact between the device case and the contralateral hand, yielding a vector that approximates the right-arm to left-arm potential difference of standard Lead I.

Each measurement comprises 60 s of raw ECG samples digitised at 600 Hz. The device uses two internal acquisition channels operated in immediate sequence; their samples are transformed to a common millivolt scale using channel-specific calibration and then assembled into a single time-series for processing. The acquisition format and transition between channels were retained as part of the native device workflow in all validation tiers. Component-level electronics, calibration coefficients, and firmware implementation details are proprietary and are not reproduced here.

### 2.2. Signal-Processing Pipeline

**Figure 2:**
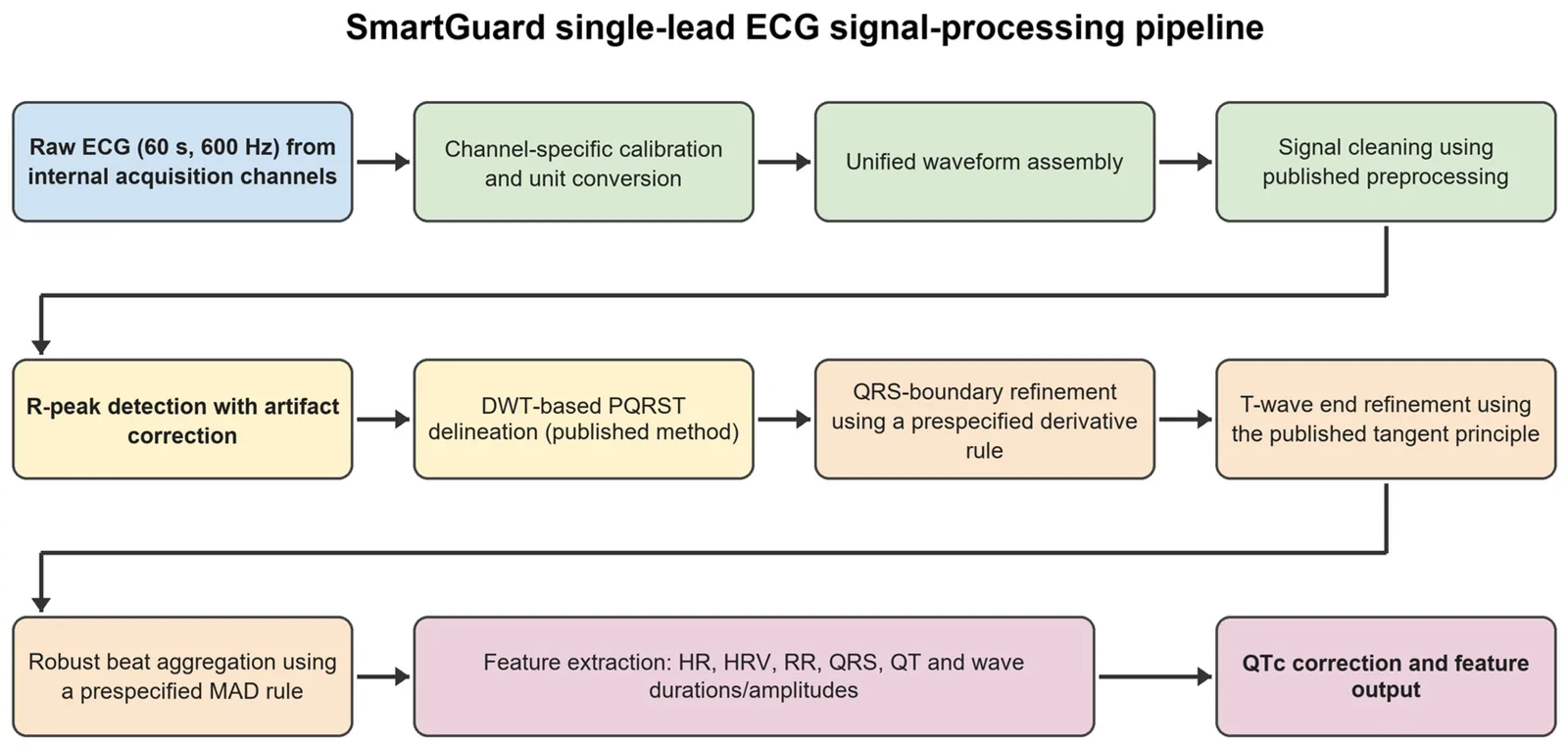
Block diagram of the SmartGuard single-lead ECG signal-processing pipeline. Raw ECG samples from the device’s internal acquisition channels are calibrated to millivolts, assembled into a unified waveform, and cleaned. R-peaks are detected with an artefact-corrected published detector, followed by discrete wavelet transform (DWT) delineation of P–Q–R–S–T fiducial points. Prespecified derivative-based QRS-boundary and tangent-based T-wave-end refinements are applied, followed by robust beat aggregation and extraction of HR, RR, QRS, QT, P/Q/R/S/T wave durations and amplitudes, and Bazett-corrected QTc. Production tuning constants are intentionally omitted. Black arrows indicate the order of processing; box colours group the stages: blue, raw input; green, calibration, assembly, and cleaning; yellow, detection and delineation; orange, prespecified refinements and robust aggregation; violet, feature extraction and output.

An overview of the signal-processing pipeline is presented in Figure 2. Each stage of the pipeline is described below. All stages were implemented in Python (version 3.10), with NeuroKit2 (version 0.2.12) [19] providing baseline cleaning, R-peak detection, and DWT-based PQRST delineation. SciPy (version 1.16.2) and NumPy (version 2.3.3) were used for supporting numerical operations.

#### 2.2.1. Cleaning and R-Peak Detection

Following calibration, the concatenated 60 s waveform is cleaned with the NeuroKit2 ecg_clean function (“neurokit” method), which applies a 0.5 Hz high-pass Butterworth filter to remove baseline wander followed by a 50 Hz notch and a powerline-safe smoothing filter [19]. R-peaks are detected with the NeuroKit2 ecg_peaks function, which combines a Pan–Tompkins-derived QRS detector [17] with an artefact-correction step that suppresses spurious detections on the basis of local RR-interval consistency. Records yielding fewer than two valid R-peaks within the 60 s window are flagged as non-analysable and excluded from downstream feature computation.

#### 2.2.2. PQRST Delineation

Fiducial points of the P, Q, R, S, and T waves (onsets, peaks, and offsets) are delineated using the discrete wavelet transform method of Martínez et al. [18], as implemented in the NeuroKit2 ecg_delineate function with method = “dwt”. DWT-based delineation provides robust automatic boundary detection across a range of morphologies and is well validated on standard public databases.

#### 2.2.3. Custom QRS-Width Refinement

DWT-derived QRS boundaries can over-estimate QRS width because the wavelet transform is sensitive to slow tails of the R-wave deflection. To address this, we implement a derivative-based QRS-width refinement. For each detected R-peak, a fixed local window is extracted from the cleaned signal, the absolute first derivative is smoothed, and its local maximum is computed. QRS onset and offset are identified by scanning outward from the principal deflection using a prespecified proportional derivative criterion. Window, smoothing, and physiological plausibility parameters were locked before the final analyses and applied unchanged across all three validation tiers; beats outside the prespecified plausible range were excluded from the per-record summary. Exact production tuning constants are maintained in controlled technical documentation and are not disclosed in the public manuscript.

#### 2.2.4. Custom T-Wave End Refinement (Tangent Method)

The end of the T wave is determined using the tangent principle originally introduced by Lepeschkin and Surawicz [20]. For each beat, a baseline reference is defined relative to the QRS complex and a local segment around the T-wave terminal limb is used to locate the steepest physiologically appropriate slope. A tangent at this point is extrapolated to the baseline to estimate the T-wave’s end. Prespecified temporal plausibility constraints are applied, and the DWT-derived T-wave end is used when the refinement does not yield a valid estimate. All settings were locked before final evaluation and held constant across datasets. The exact production timing constants are proprietary.

#### 2.2.5. Robust Averaging and Feature Extraction

**Figure 3:**
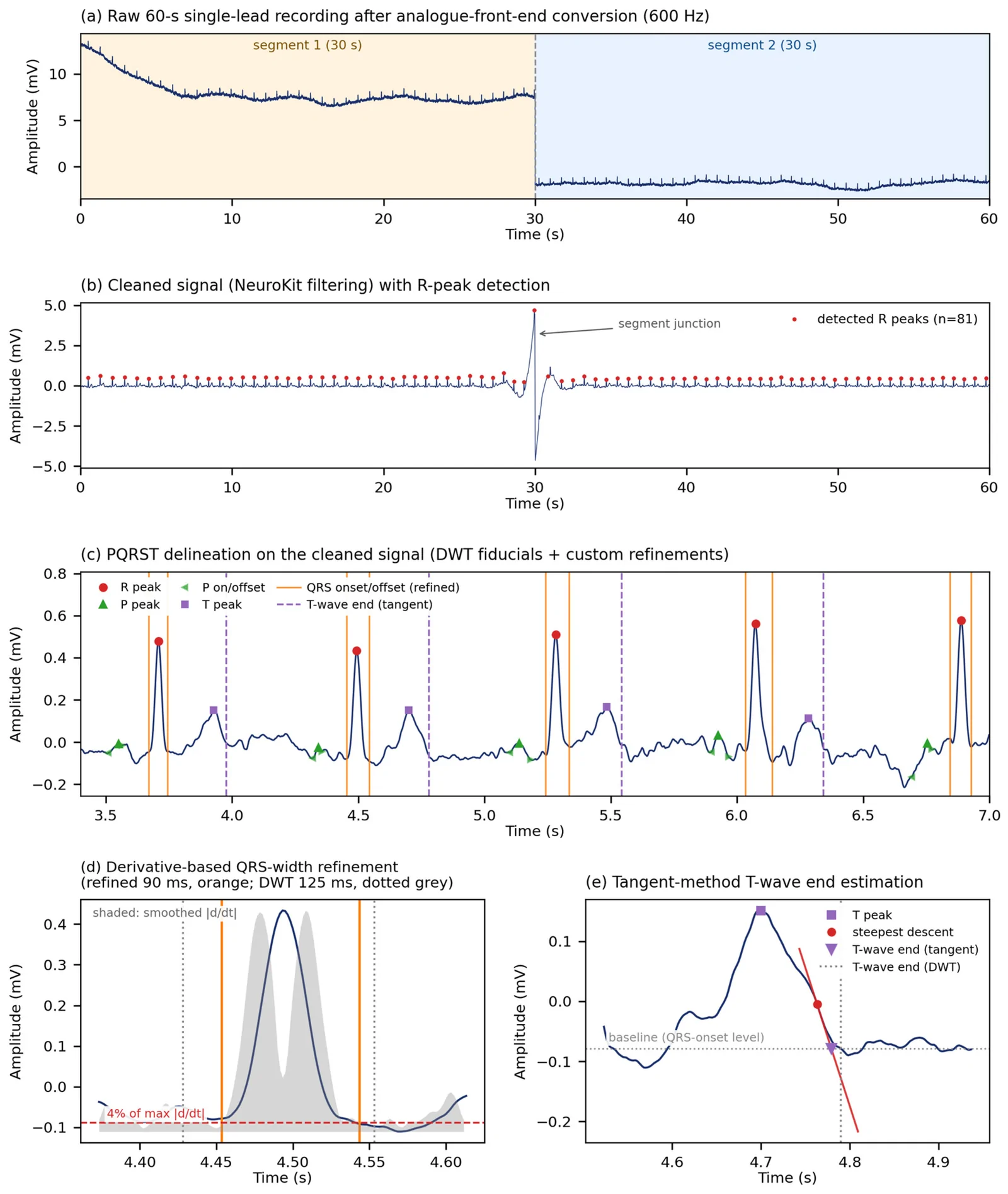
End-to-end illustration of the SmartGuard signal-processing pipeline on a representative 60-s recording from the Tier 1 study. (**a**) Raw single-lead signal after analogue-front-end conversion, showing the two consecutively acquired 30-s segments; (**b**) Cleaned signal with R-peak detection (*n* = 81; the transient at the segment junction is suppressed by artefact correction); (**c**) 3.6-s excerpt with DWT fiducials and custom refinements: P onsets/peaks/offsets, refined QRS onsets/offsets, and tangent-method T-wave ends; (**d**) Derivative-based QRS-width refinement for a representative beat—boundaries are placed where the smoothed absolute derivative falls below 4% of its local maximum, narrowing the DWT estimate from 125 ms to 90 ms (record medians 124 ms and 86 ms, respectively); (**e**) Tangent-method T-wave end—the tangent through the steepest post-peak slope is extrapolated to the baseline defined at the QRS-onset amplitude. Colour coding across panels: dark blue, ECG signal; red, R peaks, the 4% derivative threshold, and the tangent line; green, P-wave fiducials; orange, refined QRS boundaries; purple, T-wave fiducials and tangent-derived T-wave ends; dotted grey, DWT-derived reference boundaries; grey shading in (**d**), smoothed absolute derivative.

Per-beat measurements are aggregated into a single per-record summary using median-absolute-deviation (MAD) robust filtering. For each interval and amplitude metric, beats are retained using a prespecified robust deviation rule relative to the record median; the rule and cutoff were locked before final analysis and applied unchanged across all validation tiers. Surviving values are summarised by their medians for intervals or means for amplitudes. Heart rate is computed from valid RR intervals as 60/mean (RR). Corrected QT (QTc) is computed beat-by-beat using Bazett’s formula, QTc = QT/sqrt (RR), with RR in seconds, and aggregated by the same robust procedure. Per-record outputs comprise HR, HRV-RMSSD, RR, QRS, QT, QTc, and P/Q/R/S/T wave durations and amplitudes. Figure 3 provides an end-to-end illustration of every stage of this pipeline—from the raw recording through cleaning, delineation, and both custom refinements—on a representative recording from the Tier 1 study.

### 2.3. Multi-Tier Validation Framework

The pipeline was validated using a three-tier framework designed to characterise performance under (i) realistic clinical use against a contemporary reference device, (ii) cleaner ECG recordings with cardiologist-verified delineation, and (iii) longer, morphologically more variable recordings that stress the QRS- and QT-related stages of the pipeline.

#### 2.3.1. Tier 1—Within-Subject Clinical Comparison

Tier 1 was a prospective, single-centre, within-subject validation study with paired measurements. Thirty-one healthy adult volunteers (age 20–60 years; approximately 60% male, 40% female; demographic distribution kept aggregate to comply with GDPR pseudonymisation) underwent two short ECG recordings in a seated posture: one with the SmartGuard wearable ECG device in its standard Lead I-equivalent acquisition mode, and one with a Bionet Cardio 7 clinical electrocardiograph (Bionet Co., Ltd., Seoul, Republic of Korea) configured to record a Lead I-equivalent signal using only the right-arm and left-arm limb electrodes. The two-electrode limb-lead configuration was chosen specifically to match the wrist-worn device’s electrode geometry and posture as closely as possible. Recording order (watch first vs. reference first) was randomised at the participant level to minimise carryover and order effects. After a 2–3 min seated rest period, both recordings were obtained in immediate sequence without changing posture.

Inclusion criteria were age ≥ 18 and ≤60 years, ability to maintain a seated resting posture, and written informed consent. Exclusion criteria included known cardiac disease (arrhythmia, bundle-branch block, symptomatic ischaemic heart disease, heart failure), implanted cardiac electronic devices (pacemaker, ICD, CRT), pregnancy, dermatological conditions precluding safe electrode application, and inability to provide informed consent.

Reference ECG metrics—HR, QT, QTc, RR, QRS duration, and R-wave amplitude—were extracted from each Bionet Lead I trace by two independent cardiologists (with 5 and 9 years of post-specialisation experience, respectively), each blinded to the SmartGuard output. Inter-rater agreement on the manual reference measurements was 0.87 (intraclass correlation coefficient). Disagreements were resolved by consensus prior to entry into the analytic dataset. SmartGuard outputs were taken directly from the locked signal-processing pipeline described in Section 2.2 applied to the raw device signal, with no operator intervention. The study was approved by the Ethics Committee of the University of Split School of Medicine (approval number 2181-198-03-04-26-0061) and conducted in accordance with the Declaration of Helsinki and applicable Good Clinical Practice guidelines for non-interventional device validation. Written informed consent was obtained from all participants.

#### 2.3.2. Tier 2—Algorithm Benchmark on the Lobachevsky University ECG Database (LUDB)

Tier 2 compared the SmartGuard pipeline output against expert-annotated boundaries on the Lobachevsky University ECG Database (LUDB) [27]. LUDB comprises 200 ten-second 12-lead ECG recordings sampled at 500 Hz, in which boundaries and peaks of P, T, and QRS waves on each of the twelve leads were manually determined by certified cardiologists (58,429 annotated waves in total). For each LUDB record, the Lead I trace was extracted and processed by the SmartGuard pipeline at the database’s native 500 Hz sampling rate. Pipeline-derived per-record metrics (HR, HRV-RMSSD, RR, P-wave duration, QRS duration, T-wave duration, QT, QTc) were compared against ground-truth values computed from the manual cardiologist annotations using identical interval definitions. All 200 records yielded analysable signals; the analysis is therefore based on *n* = 200 paired records (with per-metric *n* reported separately where some annotations were not available—e.g., *n* = 176 for P-wave duration owing to records without delineated P-onsets). LUDB signals were processed at their native 500 Hz sampling rate; no resampling to the device’s 600 Hz was performed, as every pipeline stage defines its windows and wavelet scales in seconds and adapts them to the sampling rate of its input. Visual inspection of a subset of LUDB records informed the tuning of the custom refinement stages. However, all parameters were locked prior to the final LUDB and QTDB analyses reported here, and no record-level train–test split was performed—a design risk stated explicitly in Section 5.1.

#### 2.3.3. Tier 3—Robustness Stress-Test on the QT Database (QTDB)

**Figure 4:**
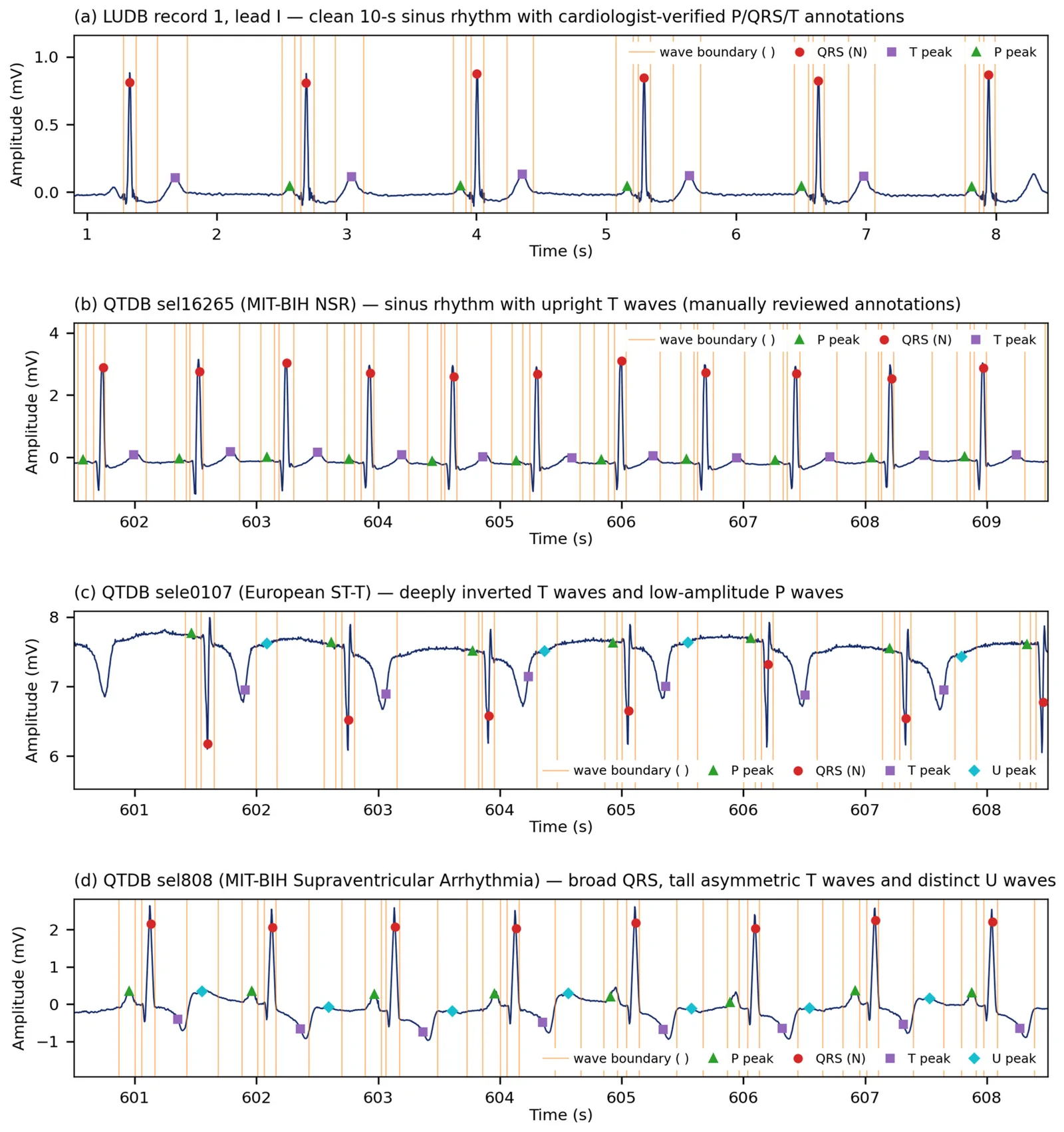
Representative expert-annotated excerpts from the two public validation databases. (**a**) LUDB record 1 (lead I): clean 10-s sinus rhythm with cardiologist-verified P (green triangles), QRS (red circles) and T (purple squares) peak annotations and wave boundaries (orange lines); (**b**) QTDB record sel16265 (MIT–BIH Normal Sinus Rhythm Database): sinus rhythm with upright T waves; (**c**) QTDB record sele0107 (European ST-T Database): deeply inverted T waves and low-amplitude P waves; (**d**) QTDB record sel808 (MIT–BIH Supraventricular Arrhythmia Database): broad QRS complexes, tall asymmetric T waves, and distinct U waves (cyan diamonds). Panels (**b**–**d**) illustrate the morphological heterogeneity that makes QTDB a deliberately harder delineation target than LUDB (Section 2.3.3). In all panels the dark-blue line is the ECG signal (amplitude in millivolts); orange vertical lines mark expert-annotated wave boundaries.

Tier 3 used the PhysioNet QT Database (QTDB) [28], which contains 105 records of variable duration drawn from multiple source databases, with manually reviewed boundary annotations on a subset of beats (the q1c expert annotation set). QTDB is widely used for validation of QT-related measurements and constitutes a deliberately more difficult test set than LUDB by virtue of longer recordings, more heterogeneous morphology, and the presence of clinical rhythm and conduction abnormalities. A hybrid gold-standard strategy was used. Manual q1c boundaries provided P-, QRS-, and QT-related ground truthing while concurrent ATR beat annotations were used where available to support RR-, HR-, HRV-, and QTc-related metrics that depend on continuous beat sequences. Records for which the gold-standard annotations did not contain at least two reference R-peaks were excluded; this resulted in 103 of the 105 records being successfully analysed. As in Tier 2, the SmartGuard pipeline was applied to a single analysis lead at the database’s native sampling rate and pipeline outputs were compared against the hybrid gold-standard reference. QTDB signals were likewise processed at their native 250 Hz sampling rate. The principal consequence of the differing sampling rates (600 Hz on-device, 500 Hz for LUDB, 250 Hz for QTDB) is the quantisation of boundary placement, which is limited to one sample period—approximately 1.7, 2.0, and 4.0 ms, respectively—so DWT-based delineation at 250 Hz carries a coarser irreducible timing granularity that contributes to the wider dispersion observed in Tier 3. Representative expert-annotated waveforms from both public databases, illustrating the contrast between the clean LUDB morphology and the heterogeneous QTDB morphologies, are shown in Figure 4.

### 2.4. Statistical Analysis

Across all three tiers, the primary analytic unit is the per-record paired difference between the SmartGuard pipeline output and the reference (clinical device or expert annotation), defined as (pipeline–reference). For each metric, we report the number of paired observations, the means of the two modalities, the mean paired difference (bias) and its standard deviation, the mean absolute error (MAE), the median percentage error, and 95% confidence intervals for the mean difference computed by standard t-based methods.

Systematic bias was evaluated using paired t-tests with a prespecified two-sided significance level of α = 0.05. For each metric, Shapiro–Wilk tests of the normality of the paired differences, together with the corresponding non-parametric Wilcoxon signed-rank tests, are reported in Supplementary Table S1, and metrics violating the normality assumption are explicitly flagged there. Agreement at the individual-pair level was characterised using Bland–Altman plots [32] showing the paired differences against within-pair means, with horizontal reference lines at the mean bias and at the upper and lower 95% limits of agreement (mean ± 1.96 × SD of the differences). Concordance between the two modalities was further summarised with Lin’s concordance correlation coefficient (CCC) [33], which captures both linear correlation and proximity to the line of equality, and with Pearson correlation coefficients for descriptive purposes. For the public-database tiers, per-record errors are additionally reported as median percentage errors to allow comparisons of relative performance across metrics of differing magnitudes. All statistical analyses were performed in Python using SciPy, NumPy, and pingouin. The Figures were produced with Matplotlib (version 3.10.6).

### 2.5. Implementation and Disclosure of Proprietary Elements

The signal-processing pipeline described in Section 2.2 was implemented in the SmartGuard device firmware and the manufacturer’s analytics environment. Its methodological foundations are published and cited: NeuroKit2 cleaning and R-peak detection [17,19], DWT-based PQRST delineation [18,19], derivative-based QRS boundary estimation derived from the Pan–Tompkins family [17], the Lepeschkin–Surawicz tangent principle for T-wave end determination [20], MAD-based robust aggregation, and Bazett QTc correction. The public description identifies the processing stages, parent methods, locked-parameter design, and validation behaviour needed to evaluate scientific validity, while omitting production tuning constants and implementation-specific decision rules.

Implementation details that are not necessary for understanding or evaluating the reported validation—including component-level calibration coefficients, on-device fixed-point implementation, low-level noise-rejection logic, auxiliary device-internal classifiers, and security-relevant production controls—are proprietary and are not reproduced here. The locked constants are maintained in controlled technical documentation and may be provided to editors or reviewers only through an appropriate confidential review process. The validation results fully characterise externally observable input/output agreement with the reference systems and expert annotations used in this study. To balance reproducibility with commercial confidentiality, Appendix A summarises, for every pipeline stage, which parameters are disclosed exactly in this paper and which are withheld, together with the admissible range or constraint rule satisfied by each withheld element. The methodological logic required for academic reproduction—cleaning, detection, delineation, refinement, and aggregation, with their governing rules and disclosed parameters—is thereby fully public. Only firmware-level implementation constants remain proprietary.

## 3. Results

All experiments follow the design summarised in Table 1. The locked pipeline of Section 2.2 was applied without modification to each tier’s input signals, and its per-record outputs were compared with the corresponding reference standard using the statistical framework of Section 2.4. The following subsections report the three tiers in turn.

### 3.1. Tier 1—Within-Subject Clinical Comparison (n = 31)

Thirty-one healthy adult volunteers contributed one analysable paired recording each, yielding 31 SmartGuard–Bionet pairs for analysis. For each participant, Lead I-equivalent measurements were obtained from the SmartGuard wearable ECG device and from the Bionet Cardio 7 reference under matched seated conditions, and within-patient paired differences were defined as (pipeline−reference). Per-metric summary statistics are presented in Table 2.

Heart rate showed essentially perfect group-level agreement (mean SmartGuard 79.07 bpm vs. Bionet 79.07 bpm; mean paired difference 0.00 ± 6.52 bpm; *p* = 1.000; 95% CI [−2.39, +2.39] bpm; Lin’s CCC 0.62), with a mean absolute error (MAE) of 4.84 bpm. The QT and QTc intervals were also unbiased on average (QT mean diff −0.60 ± 44.18 ms, *p* = 0.940; QTc mean diff −4.02 ± 54.79 ms, *p* = 0.696, *n* = 29), although both showed substantial dispersion of individual paired differences (CCC −0.06 and −0.21 respectively), consistent with known difficulties in harmonising single-lead and twelve-lead QT delineation. The RR-interval analysis closely mirrored the heart-rate result, with a mean paired difference of −0.33 ± 70.51 ms (*p* = 0.979; CCC 0.60). QRS duration showed a small positive mean difference that did not reach conventional statistical significance (mean diff +7.26 ± 21.22 ms; *p* = 0.066; CCC 0.28), suggesting modestly wider QRS estimates by the SmartGuard pipeline relative to the manual reference. R-wave peak amplitude, in contrast, was systematically underestimated by the SmartGuard relative to the Bionet (mean diff −0.15 ± 0.16 mV; *p* < 0.0001; CCC 0.13), an effect attributable to differences in gain, electrode configuration, and filtering rather than physiological variation. Inter-rater agreement of the two cardiologists who manually measured the Bionet reference traces was high (intraclass correlation coefficient 0.87), supporting the reliability of the reference-side measurements. We emphasise the correct reading of these results. The near-zero mean differences establish minimal bias at the population level only. The high dispersion of individual-record errors for QT and QTc (CCC −0.06 and −0.21) precludes accurate clinical QT interpretation from a single wearable trace in an individual patient. Furthermore, with *n* = 31 seated healthy adults, Tier 1 is designed to assess baseline bias only. It cannot establish clinical limits of agreement, does not support equivalence testing or subgroup analyses, and its findings must not be extrapolated to arrhythmic conditions or exercising states.

### 3.2. Tier 2—Algorithm Benchmark on LUDB (n = 200)

**Figure 5:**
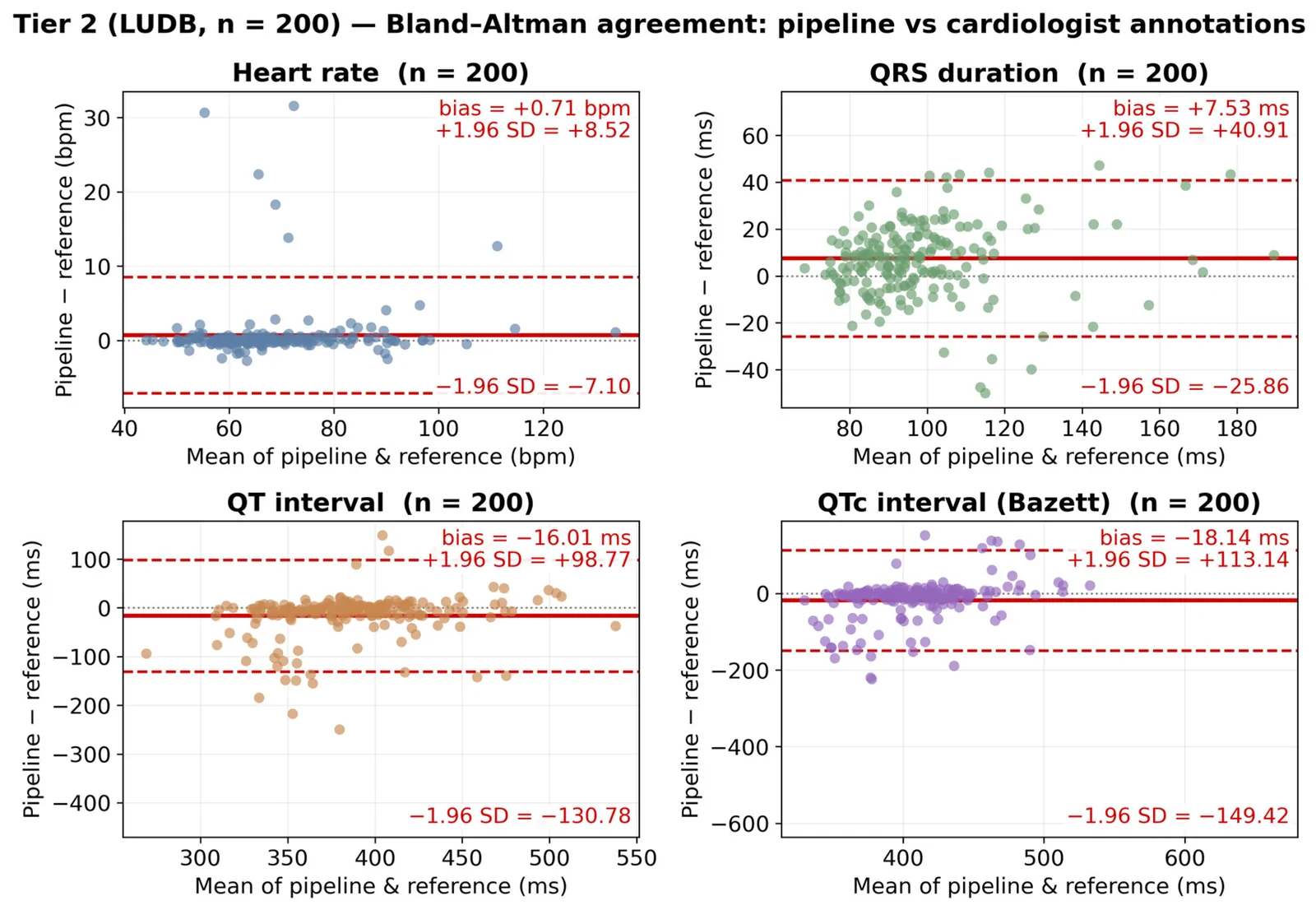
Bland–Altman plots for Tier 2 (LUDB, *n* = 200). Each scatter point represents one record. Solid red line: mean bias (pipeline−reference). Dashed red lines: upper and lower 95% limits of agreement (bias ± 1.96 × SD). Dotted grey line: zero reference. Heart rate (top-left) and QRS duration (top-right) show tight limits of agreement around small positive biases. QT (bottom-left) and QTc (bottom-right) show low aggregate bias but substantially wider limits of agreement, driven by a subset of records with discrepant T-wave end placement. Point colours identify the metric panels only (blue, heart rate; green, QRS duration; orange, QT; purple, QTc) and carry no additional meaning.

All 200 LUDB records were successfully processed by the SmartGuard pipeline (success rate 100%). Per-record agreement against the cardiologist annotations is summarised in Table 3 and visualised in Figure 5. R-peak detection and rate-related metrics performed excellently The MAE for heart rate was 1.13 bpm (bias +0.71 bpm, Pearson r = 0.955) and for the RR interval 12.01 ms (bias −7.10 ms, r = 0.957), with the 95% limits of agreement remaining tight around zero (Figure 5, top-left). HRV (RMSSD) showed good correlation (r = 0.713) with a small positive bias of +6.22 ms.

Among the delineation-dependent metrics, QRS duration showed good agreement after the derivative-based refinement described in Section 2.2.3: MAE 13.82 ms (bias +7.53 ms, r = 0.727), with the slight positive bias reflecting marginally wider QRS estimates than the cardiologist annotations. P-wave duration was estimated with moderate accuracy (MAE 14.77 ms, bias −8.24 ms, r = 0.541), while T-wave duration was systematically under-estimated by the pipeline (MAE 36.42 ms, bias −26.67 ms, r = 0.297). The QT and QTc intervals showed low aggregate bias (median percentage error around 1.4%) but substantial per-record dispersion (QT MAE 30.36 ms, r = 0.397; QTc MAE 32.83 ms, r = 0.219), consistent with the known sensitivity of single-lead QT delineation to T-wave morphology and to the precise placement of the T-wave end (Figure 5, bottom row). When interpreting the Bland–Altman panels of Figure 5, the clinically relevant quantity is the width of the 95% limits of agreement rather than the mean bias. For QT on LUDB, the limits span approximately ±115 ms around the mean difference, implying that an individual wearable-derived QT value may deviate from the annotated reference by a clinically meaningful margin even though the population-level bias is small.

### 3.3. Tier 3—Robustness Stress-Test on QTDB (n = 103)

**Figure 6:**
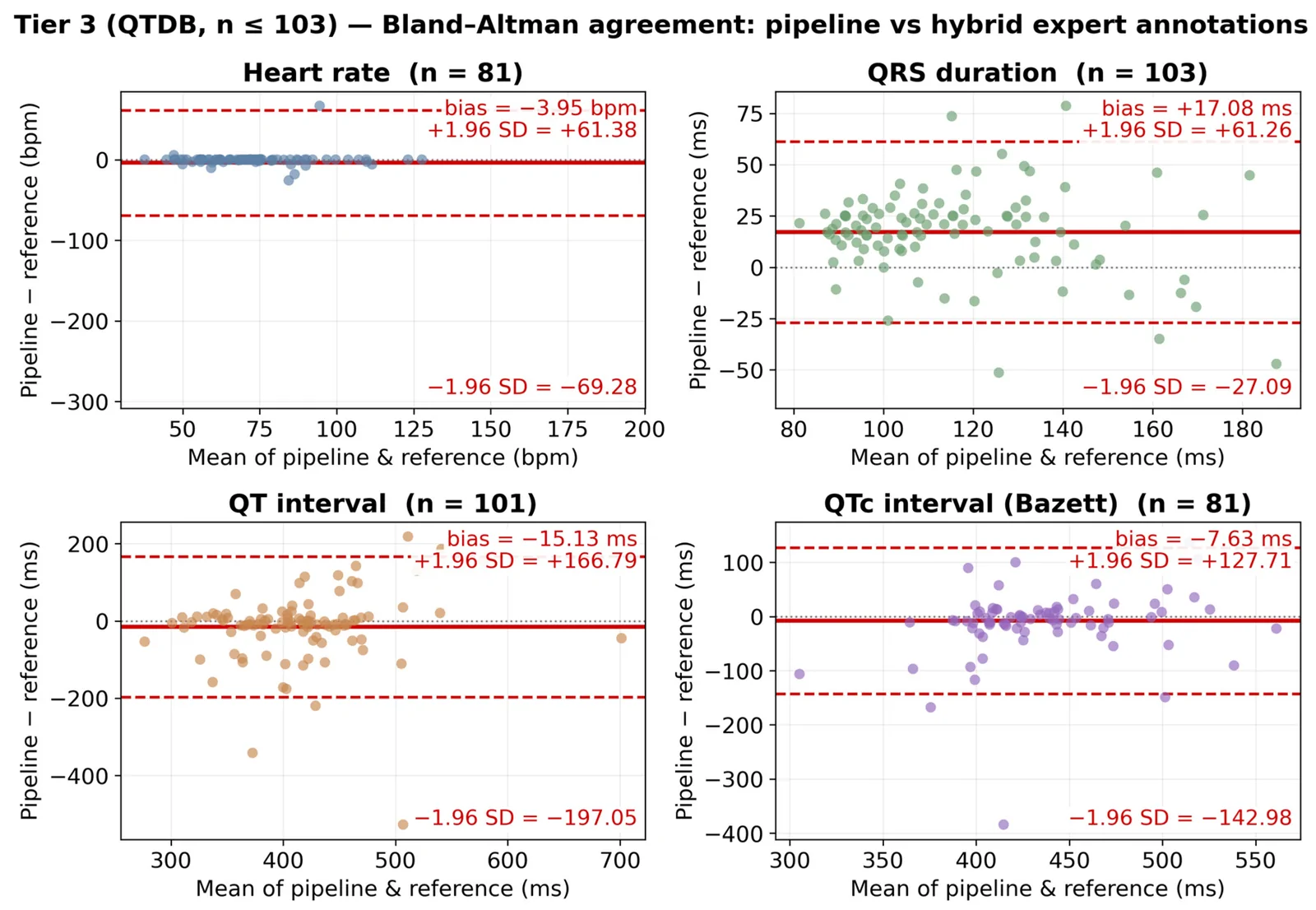
Bland–Altman plots for Tier 3 (QTDB, *n* ≤ 103). Compared with Tier 2 (Figure 5), limits of agreement are wider for all four panels—particularly for QT and QTc (bottom row)—reflecting the increased morphological complexity and longer-duration recordings of the QTDB stress-test set. The QRS duration panel (top-right) shows a clear positive bias of approximately +17 ms, indicating that the derivative-based QRS-width refinement systematically extends QRS boundaries on the heterogeneous QTDB morphologies. Representative examples of the underlying QTDB morphologies are shown in Figure 4c,d.

Of 105 available QTDB records, 103 were successfully processed by the pipeline (98.1%); 2 records (sel232 and sel36) were excluded because the gold-standard annotations contained too few reference R-peaks to compute paired metrics. QTDB results were materially weaker than the LUDB results, as expected for a stress-test set with longer recordings and more heterogeneous morphologies (Table 4, Figure 6).

In this context, “increased morphological complexity” refers to concrete properties of the QTDB recordings relative to LUDB. QTDB draws its 15-min records from seven source databases—including the MIT–BIH Arrhythmia, ST Change and Supraventricular Arrhythmia databases, the European ST-T Database, and Holter recordings of sudden-death patients—and therefore contains deeply inverted and biphasic T waves, prominent U waves that can be mistaken for T-wave activity, broad or fragmented QRS complexes, low-amplitude P waves, baseline drift, and uncalibrated Holter amplitudes. Representative examples are shown in Figure 4c,d. LUDB, by contrast, consists of 10-s resting recordings with predominantly upright, well-separated waveforms (Figure 4a). These properties directly stress the delineation subtasks that drive QT/QTc dispersion: T-peak polarity decisions, tangent-slope estimation on flattened or inverted T waves, and the separation of T-wave from U-wave activity.

The RR interval remained excellent (MAE 14.41 ms, bias −1.06 ms, r = 0.965), indicating that R-peak detection and beat-to-beat timing are robust even on morphologically variable recordings. The aggregate heart-rate MAE of 5.77 bpm is notably higher than on LUDB; this is driven by a small subset of records with extreme HR errors rather than by a uniform shift, as evidenced by a median percentage error of 0.0%. QRS duration showed a positive bias of +17.08 ms (MAE 23.63 ms, r = 0.654), indicating that the derivative-based QRS-width refinement loses some specificity on QTDB’s wider morphological range. QT and QTc interval estimation showed the largest impact: QT MAE rose to 53.0 ms (bias −15.13 ms; median percentage error −1.8%) and QTc MAE to 39.51 ms, with high standard deviations of the per-record errors (QT 92.82 ms, QTc 69.05 ms). The small median percentage errors against the larger individual-record dispersion indicates that typical QTDB cases are handled reasonably well, while a subset of difficult outlier records dominates the overall spread.

### 3.4. Cross-Tier Comparison

**Figure 7:**
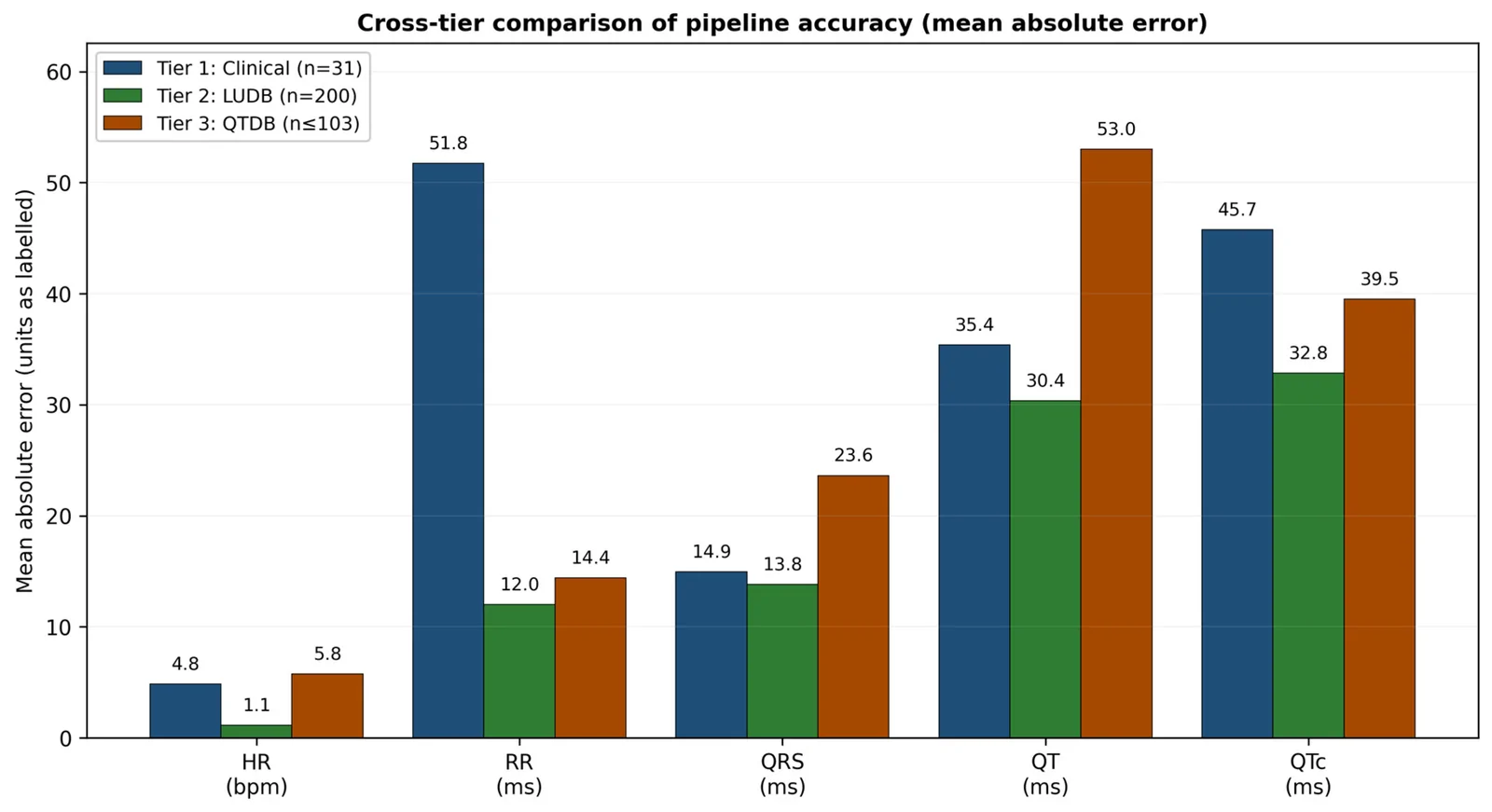
Cross-tier comparison of pipeline mean absolute error (MAE) for the five metrics most directly comparable across the three validation settings. Tier 1: Paired clinical recordings of healthy adults against the Bionet Cardio 7 Lead I reference (*n* = 31). Tier 2: Per-record comparison against cardiologist annotations on LUDB (*n* = 200). Tier 3: Per-record comparison against hybrid expert annotations on QTDB (*n* ≤ 103). Numerical values are shown above each bar.

Figure 7 and Table 5 summarise pipeline accuracy across the three validation tiers in terms of mean absolute error for the five metrics most directly comparable between the clinical and benchmark settings. The performance pattern is internally consistent. Heart rate and RR interval are most accurately recovered on the cleanest dataset (LUDB, MAE 1.13 bpm and 12.0 ms), with moderate degradation on QTDB (5.8 bpm, 14.4 ms) and on the clinical Tier 1 recordings (4.8 bpm, 51.8 ms). The larger Tier 1 RR MAE reflects that the manual reference RR was extracted from a Bionet trace rather than from per-beat algorithmic measurement, and that two short recordings—rather than a single contemporaneous trace—were compared. QRS duration shows similar MAE across Tier 1 (14.9 ms) and LUDB (13.8 ms), but a larger MAE on QTDB (23.6 ms), as expected for the stress-test set. QT and QTc accuracy follow the same ordering: best on LUDB (30.4/32.8 ms), comparable on the clinical Tier 1 (35.4/45.7 ms), and worst on QTDB (53.0/39.5 ms).

Taken together, the three tiers describe a consistent profile. The SmartGuard pipeline achieves excellent agreement for rate-related metrics (HR, RR), good agreement for QRS duration on cleaner recordings, and acceptable but morphology-sensitive performance for QT/QTc estimation. The principal residual limitations are (i) systematic underestimation of T-wave duration and consequent dispersion of QT/QTc on heterogeneous recordings, (ii) a positive QRS-duration bias on QTDB attributable to the derivative-threshold extending boundaries on morphologically wider beats, and (iii) the systematic R-wave amplitude offset against the Bionet reference identified in Tier 1, which is of calibration rather than algorithmic origin.

## 4. Discussion

### 4.1. Principal Findings

This study presents a multi-tier validation of a single-lead wearable ECG signal-processing pipeline, combining a within-subject clinical comparison (*n* = 31 healthy adults), an algorithmic benchmark on cardiologist-annotated data (LUDB, *n* = 200), and a robustness stress-test on heterogeneous expert-annotated data (QTDB, *n* = 103). Three principal findings emerge across the three tiers. First, rate-related metrics—heart rate and the RR interval—are recovered with excellent accuracy regardless of the validation tier (Pearson r ≥ 0.95 on both LUDB and QTDB; near-zero population bias on the clinical comparison), establishing that the R-peak detection and beat-to-beat timing components of the pipeline behave as intended. Second, delineation-dependent metrics (QRS duration, T-wave duration, QT and QTc) show a clear stratification by morphological complexity: agreement is good on cleaner LUDB recordings (QRS r = 0.73, MAE 13.8 ms; small QT/QTc bias of 1–2%) but degrades on the more heterogeneous QTDB stress-test set, with the QRS boundary refinement losing specificity (+17 ms positive bias) and QT estimation showing larger per-record dispersion. Third, the clinical-tier comparison reveals a small, statistically significant systematic underestimation of R-wave amplitude (−0.15 mV; *p* < 0.0001) that is consistent with calibration and electrode-geometry differences rather than algorithmic error. The internal consistency of these findings across three independent datasets supports the validity of the multi-tier framework as well as the interpretation of the individual results.

Taken together, these three findings describe a consistent gradient that is itself a useful scientific observation: temporal metrics (HR, RR) are recovered with high accuracy across all tiers, whereas morphology-dependent metrics (QRS boundaries, T-wave end, QT/QTc) degrade progressively as the dataset’s morphological heterogeneity increases. This gradient identifies where wearable-ECG pipelines remain trustworthy and where they require continued algorithmic development, and provides a structured framework for setting use-case-specific confidence thresholds rather than relying on a single global accuracy claim.

### 4.2. Comparison with Prior Wearable Single-Lead ECG Validation Studies

The pipeline’s clinical-tier performance is broadly comparable with published validation studies of contemporary single-lead and reduced-lead wearable ECG devices. In a similar study design comparing Apple Watch Lead I to a 12-lead ECG in 43 healthy volunteers, Saghir et al. reported mean differences of −6.89 ± 14.81 ms for QRS duration and −11.27 ± 22.9 ms for QT, with concordance correlation coefficients in the moderate-to-strong range [3]. Our QRS bias of +7.26 ± 21.22 ms is similar in magnitude but opposite in direction, consistent with the SmartGuard pipeline’s derivative-based QRS-width refinement extending the offset slightly further than the manual reader places it; our QT bias of −0.60 ± 44.18 ms is closer to zero on average, although the per-pair dispersion is wider. Spaccarotella et al. demonstrated that smartwatch QT estimation is lead-dependent in a 12-lead comparison [4], which is consistent with the elevated dispersion we observe for QT-related metrics in both clinical and benchmark settings. The EVALECG Cardio study compared the AliveCor KardiaMobile 6L to the standard 12-lead ECG in unselected cardiology patients and reported strong concordance for HR, PR, QRS, and QT intervals [5]; our results in healthy adults are of comparable order of magnitude, although the SmartGuard pipeline operates from a single Lead I trace rather than from six derived leads and our cohort comprises only resting healthy adults. Strik et al. similarly highlighted residual QT dispersion in remote Apple Watch monitoring [11], supporting our observation that single-lead QT estimation remains the most challenging element of the pipeline. Population-scale AF-detection studies such as the Apple Heart Study [1] and the Fitbit Heart Study [2] address a different question—binary classification of irregular rhythms—and are complementary to the quantitative-metric validation reported here rather than directly comparable.

On the algorithmic-benchmark side, our LUDB results are consistent with the literature on wavelet-based ECG delineation. Martínez et al.’s original DWT-based delineator reported sub-10 ms onset/offset errors on PhysioNet databases [18], and subsequent NeuroKit2 wrappers around this approach have performed similarly when evaluated on Lead II of LUDB [19]. Our LUDB QRS result (MAE 13.8 ms, r = 0.73) is slightly weaker than the original Martínez paper’s reported 10–11 ms onset/offset error, which is expected because (a) We evaluate on Lead I rather than the originally tested Lead II, (b) We apply an additional derivative-based QRS-width refinement that has not been individually optimised on LUDB, and (c) We operate the entire pipeline at the LUDB native 500 Hz sampling rate, slightly lower than the device’s native 600 Hz. These trade-offs are deliberate. The pipeline is designed for single-lead wrist acquisition rather than for any one benchmark database, and the LUDB and QTDB results characterise its performance under those constraints.

### 4.3. Mechanistic Interpretation of Residual Limitations

#### 4.3.1. R-Wave Amplitude and the Calibration Boundary

The most clinically interpretable finding from the clinical tier is the small but highly statistically significant underestimation of R-wave amplitude by the SmartGuard pipeline relative to the Bionet Cardio 7 reference (mean difference −0.15 ± 0.16 mV, *p* < 0.0001). The magnitude is consistent across the cohort and independent of measured QRS duration or heart rate, indicating a systematic acquisition, geometry, or filtering offset rather than a beat-by-beat algorithmic error. Plausible contributors include small differences between the device’s internally calibrated acquisition paths and the Bionet front-end, the difference between wrist-to-hand and limb-electrode vectors, and modest amplitude effects of preprocessing. The result does not affect rate-based use but establishes that absolute amplitude measures require a separate locked calibration study before use in voltage-based diagnostic criteria. The form and coefficients of any future correction are outside the scope of this validation paper.

#### 4.3.2. T-Wave End Detection and QT/QTc Dispersion

Single-lead wearable ECG systems carry less spatial information than 12-lead clinical systems. T-wave end detection is therefore expected to be more difficult. Across all tiers, T-wave end determination remains the principal source of QT/QTc dispersion. On LUDB, the pipeline under-estimates T-wave duration by approximately 27 ms (r = 0.30). On QTDB, the QT MAE is 53 ms, with a population bias of −15 ms and per-record SD of 93 ms. The tangent principle performs best on clearly terminated monophasic T waves and is challenged by low-amplitude, biphasic, notched, or U-wave-overlapping morphologies. Prespecified plausibility constraints limit extreme failure modes but cannot replace spatial information absent from a single lead. These results motivate future work on morphology- and confidence-aware robustness. Specific architectures and decision rules are reserved for separate protected development and validation.

#### 4.3.3. QRS-Width Refinement on Heterogeneous Morphologies

The fixed proportional derivative criterion used to refine QRS onset and offset trades simplicity against adaptability. On LUDB, where QRS morphologies are predominantly narrow and well formed, it delivers good agreement with cardiologist-determined boundaries (MAE 13.8 ms; bias +7.5 ms). On QTDB, where broadened and fragmented morphologies are more common, the same locked criterion extends boundaries further than the annotations (bias +17 ms; MAE 23.6 ms). Wider complexes decay more gradually, so a fixed proportional cutoff captures more of the terminal tail. The clinical tier shows an intermediate bias (+7.3 ms, *p* = 0.066) consistent with this mechanism. Future work will evaluate robustness across narrow and wide morphologies. Specific adaptive rules and calibration regimes are outside the scope of this public report. Table 6 summarises these three residual limitations, comparing each with published reference results and identifying the signal conditions that govern their magnitude.

### 4.4. Clinical Implications and Recommendations

The performance profile observed across the three tiers supports clear, use-case-specific conclusions. For applications where heart rate and RR-interval accuracy are the primary requirement—for example, ambulatory rate monitoring, screening for irregular rhythms, or fitness and recovery tracking—the SmartGuard pipeline is well-suited to the task. Performance is in the same range as the best published wearable validation studies, with MAEs comparable to or better than published 1–6 bpm ranges for HR and 10–15 ms for RR. For applications that depend on QRS duration alone in predominantly narrow-complex populations—for example, screening for intra-ventricular conduction delay above conventional thresholds (≥120 ms)—the pipeline’s MAE of around 14 ms on cleaner data is acceptable, provided the screening threshold is interpreted as a soft cut-off rather than an exact boundary. For applications that require accurate population-level QT or QTc estimation—for example, quantifying drug-induced QT prolongation in the same individual over time—the small population bias we observe is encouraging, but the per-record dispersion is large enough that clinical decision thresholds (e.g., a QTc > 480 ms threshold) should not be applied to a single SmartGuard recording without confirmation. For applications that depend on absolute amplitude criteria—for example, Sokolow–Lyon or Cornell voltage criteria for left-ventricular hypertrophy—an explicit calibration step relating SmartGuard amplitudes to a reference 12-lead system is required. The −0.15 mV systematic offset against the Bionet observed in this study provides a starting point for such a calibration. Across all use cases, the SmartGuard wearable ECG device is positioned as a complementary monitoring tool—providing stable and reproducible temporal monitoring within the known limitations of a single-lead wearable system—and is not intended to replace the diagnostic role of a clinical 12-lead ECG.

Three improvement categories follow from the results: increasing QRS-boundary robustness across heterogeneous morphologies, improving T-wave-end estimation in complex waveforms, and strengthening amplitude comparability between wearable and clinical reference systems. These are reported as validation objectives rather than implementation designs. Specific adaptation mechanisms, model architectures, routing logic, and calibration procedures are reserved for separate development, protection, and prospective validation.

### 4.5. Value of the Multi-Tier Validation Framework

The three tiers each address a question that the others cannot. The clinical comparison establishes that the pipeline behaves correctly on the device’s own raw signal under realistic resting acquisition conditions, but is small (*n* = 31), confined to healthy resting adults, and inherently limited by the precision of manual reference reading. The LUDB benchmark establishes the pipeline’s accuracy on cleanly-acquired 10 s 12-lead recordings at scale (*n* = 200), against expert-annotated boundaries, and isolates algorithmic from acquisition variability—but does so on signals that are not the SmartGuard device’s native input. The QTDB stress-test characterises pipeline robustness on longer and morphologically more diverse recordings, where conduction abnormalities and heterogeneous T-wave shapes dominate the error distribution. Read together, the three tiers support more confident interpretation than any one of them in isolation. A finding that is consistent across all three (e.g., excellent HR/RR accuracy) is unambiguously a property of the pipeline. A finding that appears only in one tier (e.g., the R-wave amplitude offset confined to Tier 1) localises the explanation to an acquisition or calibration boundary that the algorithmic tiers cannot probe. This protocol structure is generalisable to other single-lead wearable ECG devices and provides a transparent template for future validation reports. Compared with prevailing validation practice, the advantage of this design is threefold. First, it exposes failure modes that single-design studies structurally cannot detect: a clinical comparison alone cannot attribute error to a specific pipeline stage, and a database benchmark alone cannot reveal device-specific acquisition effects such as the R-amplitude calibration boundary identified in Section 4.3.1. Second, it separates analytical from clinical validity in the sense of the V3 framework [13], so that each performance claim is matched to the evidence layer that supports it. Third, because all three tiers use the same locked pipeline and a fully specified statistical plan, the entire protocol can be replicated for any other wearable single-lead ECG device at the cost of one clinical session and two public-database runs. To make the template directly actionable, a standardised operational checklist—listing, for each tier, the required datasets, reference standards, metrics, and statistical outputs—is provided as Supplementary Table S2.

### 4.6. Generalisability and Design Rationale

Several design choices in this study are likely to attract clarification questions during peer review. We address the most likely ones here for transparency.

Finally, ambulatory and exercise conditions are expected to introduce additional motion and posture artefacts that would disproportionately affect morphology-dependent metrics such as QRS-boundary placement and T-wave end detection. Rate-related metrics, which are recovered robustly even in our heterogeneous-morphology stress-test set, are likely to remain the most reliable outputs in such conditions. Dedicated ambulatory validation, ideally including controlled motion artefact and exercise-induced morphology changes, is a clear next step before the pipeline’s morphology-dependent outputs are used in those settings.

Motion artefacts were intentionally minimised in the present clinical tier by use of a seated resting protocol with a short rest period and explicit instructions to minimise movement during acquisition. This was a deliberate choice for a first-line validation aimed at characterising the pipeline’s behaviour under controlled conditions. Quantifying the effect of motion artefacts and posture variability on pipeline outputs is a question for subsequent ambulatory work rather than for the present manuscript. We regard motion-artefact validation as the highest-priority follow-up, with two concrete protocols: (i) controlled noise-injection experiments in which calibrated baseline-wander, muscle-artefact, and electrode-motion noise templates from the MIT–BIH Noise Stress Test Database are added to Tier 1 recordings at graded signal-to-noise ratios, quantifying the degradation of QRS and QT/QTc accuracy as a function of noise type and level; and (ii) a prospective ambulatory session in which SmartGuard recordings are acquired during standardised activities (walking, stair climbing, simulated daily tasks) with a synchronised reference ECG, enabling paired-difference analysis under genuine motion.

The two public-database tiers were chosen for their complementarity rather than their representativeness of wearable acquisition conditions. QTDB in particular comprises older clinical recordings with morphologies and noise characteristics that are not necessarily representative of wrist-worn ECG signals; it is used here specifically as a deliberate stress-test of pipeline robustness to morphological heterogeneity, rather than as a proxy for ambulatory wearable performance. The ordering Tier 1 → Tier 2 → Tier 3 should therefore be understood as a progression in algorithmic difficulty, not as a progression toward real-world wearable conditions.

Tier 1 was conducted in healthy adult volunteers under controlled resting conditions to provide a clean first-line characterisation of pipeline behaviour against a clinical reference, free of confounding from arrhythmia, conduction abnormalities, or motion artefacts. This design choice is consistent with the staged validation approach adopted by other contemporary wearable ECG validation studies [3,5] and reflects the practical scope of a first publication. Extension to patient cohorts (including individuals with conduction disease, arrhythmia, or cardiac comorbidity) is a separate validation question and is reserved for follow-up work.

We selected LUDB and QTDB as the two public-database tiers because each addresses a distinct validation question that is not well covered by the other. LUDB provides 200 cardiologist-annotated 10 s 12-lead recordings with manually verified P, QRS, and T boundaries [27], which makes it the cleanest available benchmark for single-lead delineation performance under high-quality acquisition. QTDB [28] provides longer recordings of varied morphology drawn from multiple source databases with expert q1c annotations, which makes it the canonical stress-test for QT-interval robustness; it has been used for exactly this purpose in much of the published QT-delineation literature. Both databases are openly available on PhysioNet, which supports full reproducibility of the analyses reported in this manuscript.

## 5. Limitations and Conclusions

### 5.1. Limitations

This study has several limitations that should be borne in mind when interpreting the results. The clinical-tier validation was conducted at a single centre on a convenience sample of 31 healthy adult volunteers aged 20–60 years recorded under controlled, seated, resting conditions. The sample is sufficient for reliable estimation of mean within-subject biases and basic 95% confidence intervals, but is not powered to establish narrow limits of agreement for individual-level clinical decisions, to support formal equivalence testing using two one-sided tests and prespecified margins, or to permit detailed subgroup analyses by age, sex, body habitus, or skin tone. The cohort excluded children, elderly participants, and individuals with known cardiac disease or with implanted cardiac electronic devices. Performance under arrhythmia, ischaemic, ambulatory, or exercise conditions therefore remains formally untested in the present work, and the results should not be extrapolated to those settings without further dedicated validation. In particular, the Tier 1 sample of 31 seated healthy adults—excluding patients with cardiac conditions, older adults, and children—is suitable only for assessing baseline bias. It cannot establish clinical limits of agreement, and no equivalence testing or subgroup analysis is statistically supportable at this sample size. Future work should expand the cohort to include participants with cardiovascular disease across diverse age strata.

Several methodological limitations apply to the reference standard and the validation infrastructure. The Bionet Cardio 7 reference was configured in a Lead I-equivalent two-electrode limb-lead arrangement to match the wrist-worn device’s electrode geometry, rather than as a full diagnostic 12-lead ECG. This constrains the clinical reference to a single vector and limits the depth of comparison for delineation-dependent metrics that benefit from multi-lead disambiguation. The reference measurements were extracted by two independent cardiologists, with inter-rater agreement reported here as an intraclass correlation coefficient of 0.87. The PhysioNet QT Database used in Tier 3 contains older recordings of variable signal quality and morphology, and a small subset of records dominates the per-record error distribution rather than a uniform shift across the dataset; we report median percentage errors alongside MAE specifically to make this distributional structure visible. We also underline that the Bionet Cardio 7 reference was operated in a two-electrode limb configuration approximating Lead I. This deliberately matches the lead geometry of the wearable—isolating processing differences from lead-placement differences—but forgoes the multi-lead information that a full diagnostic 12-lead ECG provides for waveform delineation. These results must therefore not be read as validation against a complete 12-lead gold standard.

The pipeline was developed iteratively, and the custom QRS, QT, and robust-aggregation parameters were guided in part by visual inspection of LUDB recordings, as documented in Section 2.2.3. The complete parameter set was locked before the final analyses and applied unchanged to the full LUDB benchmark and the independent QTDB stress-test. Because LUDB was not divided into development and held-out subsets, formal cross-validation and external generalisation on additional independent datasets would further reduce the residual overfitting risk. No amplitude-correction model was fitted in this study. Any such model would require a separately specified development set and independent prospective replication. Because visual inspection of LUDB records informed parameter tuning and no train–test split was performed, a mild risk of over-fitting to LUDB cannot be excluded. All parameters were locked prior to the final analyses, but external validation on fully unseen, independent ECG datasets is still recommended. A formal sensitivity analysis quantifying performance shifts under perturbations of the key thresholds (e.g., the proportional derivative threshold and the MAD cut-off) is a further worthwhile extension. Qualitatively, small threshold perturbations move QRS boundaries continuously rather than abruptly, so graceful rather than catastrophic degradation is expected.

### 5.2. Conclusions

We have presented a multi-tier validation of a single-lead wearable ECG signal-processing pipeline that combines clinical comparison against a limb-lead Lead I reference (*n* = 31 healthy adults), algorithmic benchmarking on cardiologist-annotated data (LUDB, *n* = 200), and a robustness stress-test on heterogeneous expert-annotated data (QTDB, *n* = 103). Across all three tiers, the pipeline recovers heart rate and the RR interval with excellent accuracy (Pearson r ≥ 0.95 on both database benchmarks, and negligible mean bias against the clinical reference). QRS-duration agreement is good on cleaner data and degrades systematically on heterogeneous morphologies, in a manner consistent with the fixed proportional derivative threshold used to refine QRS boundaries. QT and QTc estimation show low population bias but substantial per-record dispersion, dominated by the difficulty of placing the T-wave end accurately on a single lead. An additional, statistically significant systematic underestimation of R-wave amplitude relative to the clinical reference is consistent with calibration- and electrode-geometry-related differences rather than algorithmic error.

Taken together, the results support use of the SmartGuard pipeline for rhythm- and rate-based applications and identify three broad priorities for further work: QRS-boundary robustness, T-wave-end robustness, and amplitude comparability. The specific technical solutions remain outside the scope of this public validation report. Beyond the evaluated device, the multi-tier protocol—clinical comparison, expert-database benchmarking, and an explicit stress-test set—is generalisable to other single-lead wearable ECG devices and provides a transparent template for future validation reports.

## Figures and Tables

**Table 1:** Overview of the three-tier experimental design. Each tier confronts the same locked signal-processing pipeline with a different reference standard.

Tier	Dataset (*n*)	Reference Standard	Metrics Compared	Purpose
Tier 1—clinical comparison	31 healthy adults; paired 60-s SmartGuard vs. Bionet Cardio 7 recordings, matched posture	Bionet Lead I traces; manual measurements by two independent cardiologists	HR, RR, QT, QTc, QRS duration, R-wave amplitude	Clinical validation under realistic acquisition conditions
Tier 2—algorithm benchmark	LUDB; 200 ten-second records (Lead I extracted)	Cardiologist-annotated P, QRS, and T boundaries	HR, RR, HRV (RMSSD), P/QRS/T durations, QT, QTc	Analytical validation under clean, standardised conditions
Tier 3—robustness stress-test	QTDB; 103 of 105 fifteen-minute records analysed	Manually reviewed q1c annotations (hybrid gold standard)	HR, RR, QRS duration, T duration, QT, QTc	Analytical validation under deliberately heterogeneous morphology

**Table 2:** Tier 1 clinical comparison (*n* = 31 healthy adults). Pairwise statistics for the SmartGuard pipeline against the Bionet Cardio 7 Lead I-equivalent reference. Differences are computed as (pipeline − reference). MAE = mean absolute error; CI = confidence interval; CCC = Lin’s concordance correlation coefficient.

Metric	*n*	Mean (Watch)	Mean (Ref.)	Mean Diff	SD Diff	95% CI	*p*-Value	MAE	CCC
HR (bpm)	31	79.07	79.07	0.00	6.52	−2.39 to +2.39	1.000	4.84	0.62
QT (ms)	31	332.5	333.1	−0.60	44.18	−16.80 to +15.61	0.940	35.37	−0.06
QTc (ms)	29	376.2	380.2	−4.02	54.79	−24.86 to +16.82	0.696	45.74	−0.21
RR (ms)	31	763.6	764.0	−0.33	70.51	−26.19 to +25.53	0.979	51.75	0.60
QRS (ms)	31	77.10	69.84	+7.26	21.22	−0.52 to +15.04	0.066	14.94	0.28
R-amp (mV)	31	0.56	0.71	−0.15	0.16	−0.21 to −0.09	<0.0001	0.18	0.13

**Table 3:** Tier 2 LUDB benchmark (*n* = 200). Per-record agreement of pipeline outputs against cardiologist-annotated ground truth on Lead I.

Metric	*n*	MAE	Bias	Std	Median % Error	Pearson r
HR (bpm)	200	1.13	+0.71	3.99	0.0%	0.955
RR (ms)	200	12.01	−7.10	45.49	0.0%	0.957
HRV/RMSSD (ms)	200	21.91	+6.22	78.26	−3.2%	0.713
P-wave duration (ms)	176	14.77	−8.24	19.73	−2.4%	0.541
QRS duration (ms)	200	13.82	+7.53	17.03	+8.6%	0.727
T-wave duration (ms)	200	36.42	−26.67	33.72	−17.4%	0.297
QT interval (ms)	200	30.36	−16.01	58.56	−1.4%	0.397
QTc (Bazett) (ms)	200	32.83	−18.14	66.98	−1.4%	0.219

**Table 4:** Tier 3 QTDB stress-test (*n* = 103 of 105 records analysed). Per-record agreement of pipeline outputs against the hybrid manual q1c plus ATR expert annotations.

Metric	*n*	MAE	Bias	Std	Median % Error	Pearson r
HR (bpm)	81	5.77	−3.95	33.33	0.0%	0.352
HRV/RMSSD (ms)	81	31.62	−24.33	58.21	−13.7%	0.716
P-wave duration (ms)	96	20.43	−14.94	26.90	−7.2%	0.190
QRS duration (ms)	103	23.63	+17.08	22.54	+20.3%	0.654
RR (ms)	81	14.41	−1.06	58.88	0.0%	0.965
QT interval (ms)	101	53.00	−15.13	92.82	−1.8%	0.293
QTc (Bazett) (ms)	81	39.51	−7.63	69.05	−1.0%	0.247

**Table 5:** Cross-tier MAE summary for the five metrics shown in Figure 7.

Metric (Units)	Tier 1: Clinical (*n* = 31)	Tier 2: LUDB (*n* = 200)	Tier 3: QTDB (*n* ≤ 103)
HR (bpm)	4.84	1.13	5.77
RR (ms)	51.75	12.01	14.41
QRS (ms)	14.94	13.82	23.63
QT (ms)	35.37	30.36	53.00
QTc (ms)	45.74	32.83	39.51

**Table 6:** Residual limitations in context: per-tPer SmartGuard findings compared with published reference studies, together with the signal conditions that govern each limitation. Values are drawn from Section 3, Section 4.2 and Section 4.3.

Limitation Domain	This Study (per Tier)	Published Comparison	Governing Condition
R-wave amplitude	Tier 1: −0.15 ± 0.16 mV vs. Bionet reference (*p* < 0.0001); consistent across the cohort, independent of QRS duration and heart rate	Published wearable validations report interval metrics primarily; no directly comparable amplitude bias available [3,4,5]	Systematic acquisition/calibration offset (front-end paths, electrode vector), not a beat-level algorithmic error; voltage-based criteria require a locked calibration study first
QRS duration	Tier 1: +7.26 ± 21.22 ms; Tier 2 (LUDB): bias +7.5 ms, MAE 13.8 ms; Tier 3 (QTDB): bias +17 ms, MAE 23.6 ms	Apple Watch vs. 12-lead: −6.89 ± 14.81 ms [3]; original DWT delineator: ≈10–11 ms boundary error on PhysioNet databases [18]	Fixed 4% proportional derivative cut-off captures more of the terminal tail as QRS morphology broadens (clean LUDB → heterogeneous QTDB)
QT/QTc interval	Tier 1 (QT): −0.60 ± 44.18 ms; Tier 2: MAE 30.4 ms; Tier 3: MAE 53.0 ms, per-record SD 93 ms	Apple Watch QT: −11.27 ± 22.9 ms [3]; lead-dependent smartwatch QT agreement [4]; residual QT dispersion in remote monitoring [11]	Dispersion grows with morphological heterogeneity while population-level bias stays small in all tiers; individual-trace QT reading is precluded (Section 3.1)
T-wave end	Tier 2: T-duration bias ≈ −27 ms (r = 0.30); principal source of QT/QTc dispersion in every tier	Tangent-method accuracy has been morphology-dependent since its introduction [20]	Optimal on clearly terminated monophasic T waves; degraded by low-amplitude, biphasic, notched, or U-wave-overlapping morphologies (Figure 4c,d)

## Data Availability

LUDB and QTDB are publicly available on PhysioNet (https://physionet.org). Pseudonymised per-record comparisons generated from these public datasets and the statistical analysis scripts used to reproduce the reported summary statistics and figures may be made available by the corresponding author on reasonable request, subject to applicable policies. Production signal-processing source code, device firmware, proprietary calibration coefficients, tuning constants, internal classifier logic, and security-relevant implementation details are not included in the shared materials. The Tier 1 dataset contains pseudonymised physiological measurements; aggregate results are reported in full, and per-subject data may be shared only following GDPR review and institutional approval. Confidential materials—including exact firmware calibration constants and the quality-gate decision logic—can be made available to the editors and reviewers for confidential inspection upon reasonable request, subject to a non-disclosure agreement with the manufacturer.

## Supplementary Materials

The following supporting information can be downloaded at: https://www.mdpi.com/article/10.3390/s26185905/s1: Table S1: Shapiro–Wilk normality assessment of per-record paired differences (pipeline − reference) and Wilcoxon signed-rank tests, alongside the parametric paired t-tests, for the Tier 2 (LUDB) and Tier 3 (QTDB) analyses. Bias and MAE in the metric’s native unit (bpm for heart rate, ms otherwise). The Shapiro–Wilk test rejects normality (*p* < 0.05) for every metric in both tiers; the Wilcoxon signed-rank test is therefore the more appropriate test of systematic bias throughout, and the parametric *p*-values are given for completeness only. Discordant cases (e.g., heart rate and RR interval on LUDB, where the t-test suggests bias but the Wilcoxon test does not) reflect heavy-tailed difference distributions in which a small number of outlying records dominate the mean; Table S2: Standardised operational checklist for applying the three-tier validation template to another wearable single-lead ECG device.

## Appendix A

This appendix delineates the boundary between the publicly reproducible methodology and the proprietary firmware implementation. Table A1 lists, for each pipeline stage, the parameters disclosed exactly in this manuscript, the elements withheld as commercial-in-confidence, and the admissible range or constraint rule that each withheld element satisfies. All methodological logic required for academic reproduction is public; withheld elements concern only firmware-level implementation.

**Table A1:** Disclosure map of pipeline parameters: public methodology versus withheld firmware implementation details.

Pipeline Stage	Disclosed Exactly in this Manuscript	Withheld Element	Constraint/Admissible Range
Cleaning and R-peak detection	NeuroKit2 ecg_clean (“neurokit” method) and ecg_peaks with artefact correction [17,19]	None—fully public	—
PQRST delineation	DWT delineator (“dwt” method) [18,19]	None—fully public	—
QRS-width refinement	Proportional derivative threshold (4% of local |d/dt| maximum); ±120 ms search window; 30–250 ms acceptance range	Fixed-point scaling of the derivative computation in firmware	Power-of-two quantisation; must not shift boundaries by more than one sample period
T-wave end refinement	Tangent method; baseline at QRS-onset amplitude; clamps of ≤400 ms after the T peak and ≤700 ms after QRS onset	Firmware smoothing-kernel implementation	Short moving-average kernels (3–5 samples)
Robust aggregation	MAD filtering with threshold 3.5; median aggregation; 180–700 ms QT acceptance range	None—fully public	—
Analogue front-end conversion	Two consecutive 30-s segments at 600 Hz; conversion to millivolts (Section 2.1)	Sensor-specific calibration constants (gain, offset)	Affine (linear) mapping to mV; constants fixed per hardware revision
Quality gating	Existence and role disclosed (Section 2.5)	Gate decision logic and thresholds	Gate may only reject segments; it cannot alter metric values
