# Supplementary material for: Multi-Tier Validation of a Single-Lead Wearable ECG Signal-Processing Pipeline: Clinical Comparison and Algorithmic Benchmarking on Public Expert-Annotated Databases
Source: Sensors (Basel). 2026 Sep 17;26(18):5905. doi: 10.3390/s26185905 (PMC13611804; doi:10.3390/s26185905)
Supplement: Supplementary file 1 [file sensors-26-05905-s001.zip › sensors-4504469-supplementary.pdf]

Supplementary Materials

Multi-Tier Validation of a Single-Lead Wearable ECG Signal-Processing Pipeline: Clinical Comparison and Algorithmic Benchmarking on Public Expert-Annotated Databases

Josip Vrdoljak and Dora Racz

Table S1. Shapiro–Wilk normality assessment of per-record paired differences (pipeline − reference) and Wilcoxon signed-rank tests, alongside the parametric paired t-tests, for the Tier 2 (LUDB) and Tier 3 (QTDB) analyses. Bias and MAE in the metric's native unit (bpm for heart rate, ms otherwise). The Shapiro–Wilk test rejects normality ($p < 0.05$) for every metric in both tiers; the Wilcoxon signed-rank test is therefore the more appropriate test of systematic bias throughout, and the parametric p-values are given for completeness only. Discordant cases (e.g., heart rate and RR interval on LUDB, where the t-test suggests bias but the Wilcoxon test does not) reflect heavy-tailed difference distributions in which a small number of outlying records dominate the mean.

Tier	Metric	n	Bias	MAE	Shapiro–Wilk p	Normal?	Wilcoxon p	t-test p
Tier 2 (LUDB)	Heart rate (bpm)	200	0.71	1.13	7.3×10^{-27}	violated	0.344	0.012
Tier 2 (LUDB)	HRV / RMSSD (ms)	200	6.22	21.91	2.5×10^{-26}	violated	0.051	0.262
Tier 2 (LUDB)	P-wave duration (ms)	176	-8.24	14.77	4.0×10^{-7}	violated	1.4×10^{-5}	1.1×10^{-7}
Tier 2 (LUDB)	QRS duration (ms)	200	7.53	13.82	4.4×10^{-5}	violated	1.5×10^{-10}	2.5×10^{-9}
Tier 2 (LUDB)	T-wave duration (ms)	200	-26.67	36.42	8.4×10^{-11}	violated	5.7×10^{-20}	7.5×10^{-23}
Tier 2 (LUDB)	RR interval (ms)	200	-7.10	12.01	2.1×10^{-27}	violated	0.154	0.028
Tier 2 (LUDB)	QT interval (ms)	200	-16.01	30.36	1.2×10^{-18}	violated	7.5×10^{-6}	1.5×10^{-4}
Tier 2 (LUDB)	QTc, Bazett (ms)	200	-18.14	32.83	3.3×10^{-20}	violated	8.1×10^{-7}	1.7×10^{-4}
Tier 3 (QTDB)	Heart rate (bpm)	81	-3.95	5.77	3.7×10^{-19}	violated	4.9×10^{-4}	0.290
Tier 3 (QTDB)	HRV / RMSSD (ms)	81	-24.33	31.62	1.4×10^{-11}	violated	8.7×10^{-9}	3.2×10^{-4}
Tier 3 (QTDB)	P-wave duration (ms)	96	-14.94	20.43	5.8×10^{-7}	violated	7.5×10^{-8}	4.1×10^{-7}
Tier 3 (QTDB)	QRS duration (ms)	103	17.08	23.63	7.6×10^{-5}	violated	4.7×10^{-11}	9.4×10^{-12}
Tier 3 (QTDB)	RR interval (ms)	81	-1.06	14.41	7.3×10^{-18}	violated	0.079	0.872
Tier 3 (QTDB)	QT interval (ms)	101	-15.13	53.00	3.5×10^{-10}	violated	0.071	0.104
Tier 3 (QTDB)	QTc, Bazett (ms)	81	-7.63	39.51	4.0×10^{-9}	violated	0.233	0.323

Note: Tier 1 (clinical) inferential statistics are reported in the main text (Section 3.1); per-record clinical differences are held with the study dataset under the conditions stated in the Data Availability Statement.

Table S2. Standardised operational checklist for applying the three-tier validation template to another wearable single-lead ECG device.

Checklist item	Tier 1 — clinical comparison	Tier 2 — algorithm benchmark	Tier 3 — robustness stress-test
Purpose	Clinical validation under realistic acquisition	Analytical validation under clean conditions	Analytical validation under morphological heterogeneity
Dataset	Prospective paired recordings; device + reference in matched posture; predefined inclusion/exclusion criteria; ethics approval and informed consent	Expert-annotated public database with clean, standardised recordings (e.g., LUDB)	Expert-annotated public database with heterogeneous morphology and longer recordings (e.g., QTDB)
Sample size	Report n and justify; state explicitly whether the design supports bias assessment only or also limits of agreement / equivalence	All records of the chosen database; report per-metric n where annotations are missing	All records processable; report and justify exclusions
Reference standard	Simultaneous or immediately adjacent clinical ECG; manual measurement by ≥2 independent readers; report inter-reader agreement	Database expert annotations (peak + boundary level)	Manually reviewed annotations; document hybrid/gold-standard construction
Signal handling	Lock the pipeline before analysis; process device output without operator intervention	Extract the device-equivalent lead; process at native sampling rate; document any resampling	Same as Tier 2; document sampling-rate consequences for boundary quantisation
Metrics	HR, RR, QT, QTc, QRS duration, amplitudes as applicable	All delineation-dependent metrics the pipeline outputs	Subset of Tier 2 metrics supported by the annotations
Statistics	Paired differences: bias ± SD, MAE, 95% CI, paired t-test AND Wilcoxon (report normality tests), Bland–Altman with LoA, CCC, Pearson r	Same as Tier 1, plus median percentage error per metric	Same as Tier 2; compare dispersion against Tier 2 to quantify degradation
Reporting outputs	Per-metric table; Bland–Altman plots; explicit population-level vs individual-level interpretation	Per-metric table; Bland–Altman plots; success rate	Per-metric table; Bland–Altman plots; excluded-record listing with reasons
Failure-mode analysis	Attribute residual biases to acquisition vs processing (e.g., calibration boundary)	Attribute errors to delineation subtasks (boundary conventions, T-end)	Identify morphology classes driving degradation; link to concrete algorithm remedies
